# Review: Application of Nanoparticles in Urothelial Cancer of the Urinary Bladder

**DOI:** 10.1007/s40846-015-0060-5

**Published:** 2015-08-11

**Authors:** Chieh-Hsiao Chen, Tzu-Min Chan, Yi-Jhen Wu, Jia-Jin Chen

**Affiliations:** Institute of Biomedical Engineering, National Cheng Kung University, 1 University Road, Tainan, 701 Taiwan; Department of Urology, China Medical University Beigang Hospital, 123 Sin-Der Road, Beigang, 651 Yunlin Taiwan; Department of Medical Education and Research, China Medical University Beigang Hospital, 123 Sin-Der Road, Beigang, 651 Yunlin Taiwan

**Keywords:** Urothelial cancer, Nanoparticles, Nanotechnology, Photothermal Therapy, Drug delivery

## Abstract

Bladder cancer is a common malignancy of the urinary tract, which generally develops in the epithelial lining of the urinary bladder. The specific course of treatment depends on the stage of bladder cancer; however, therapeutic strategies typically involve intravesical drug delivery to reduce toxicity and increase therapeutic effects. Recently, metallic, polymeric, lipid, and protein nanoparticles have been introduced to aid in the treatment of bladder cancer. Nanoparticles are also commonly used as pharmaceutical carriers to improve interactions between drugs and the urothelium. In this review, we classify the characteristics of bladder cancer and discuss the types of nanoparticles used in various treatment modalities. Finally we summarize the potential applications and benefits of various nanoparticles in intravesical therapy.

## Introduction

Urothelial cancer of urinary bladder is an epithelial cancer in which abnormal cells in the epithelial lining multiply without control [1, 2]. The most common type of bladder cancer is transitional cell carcinoma (TCC), also referred to as urothelial cell carcinoma (UCC). Over the last two decades, various nanoparticle technologies have been used in the detection and treatment of cancers of the breast [3], oral cavity [4], lung [5], cervix [6], and brain [7]. Nanoparticles are also used as pharmaceutical carriers in drug delivery systems comprising organic and inorganic materials [8, 9], and many state-of-the-art techniques incorporate liposomal [10], polymer-drug conjugates [11] and micellar formulations [12]. Furthermore, a considerable number of nanoparticle platforms are currently in the preclinical stages of development [13]. In this review, we classify the various types of bladder cancer according to their clinical characteristics and summarize how various nanoparticles are applied in intravesical bladder cancer therapy. This work is of particular importance at this time, due to recent findings showing that the use of nanoparticles in the treatment of urothelial cancer in the urinary bladder can reduce negative side effects and recurrence rates.

## Current Treatment of Urothelial Cancer of the Urinary Bladder

Bladder carcinomas the fourth most common malignancy in America and the fifth most common disease among European males [14]. Furthermore, the prevalence of this malignancy of the urinary tract tends to increase with economic development [15–17]. The most typical symptom of this malignancy is painless hematuria, with microscopic or gross hematuria presenting in more than 85 % of patients [18]. Depending on the severity of hemorrhaging, the color of urine can range from normal, to dark yellow, to bright red or cola [19]. Other symptoms include increased frequency and urgency of urination, dysuria, and abdominal pain [20]. The five-year average survival rate among patients with bladder cancer is approximate 60 %; however, bladder cancer is associated with a high recurrence rate, which results in a longer course of disease with greater per-patient financial cost compared to other cancers [21].

**Fig. 1:**
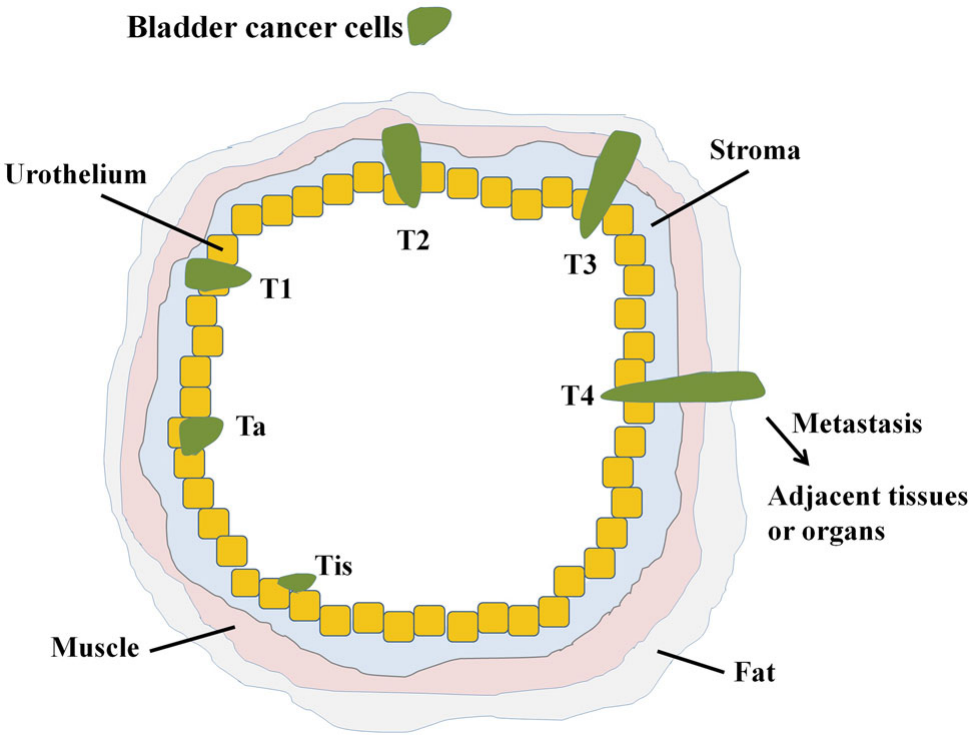
A schematic diagram illustrating the classification of bladder cancer: *Tis* carcinoma in situ (‘flat tumour’); *T1* tumour invades subepithelial connective tissue; *T2* tumour invades muscle; *T3* tumour invades perivesical tissue; *T4* tumour invades prostate, uterus, or vagina

The bladder is a hollow, distensible organ used for the storage of urine [22]. It is composed of mucosa, submucosa, detrusor muscle, and perivesical fat. Bladder cancer typically begins in the mucosa layer, namely, transitional epithelial tissue [23]. TCC comprises over 90 % of bladder cancers; other bladder cancer types include squamous cell carcinoma (SCC, about 7–8 %), adenocarcinoma (1–2 %), and carcinosarcoma (<1 %) [24]. Furthermore, most diagnosed cases (70–85 %) involve a superficial (non-muscle invasive) form of the disease [25–27]. Bladder cancer staging is classified according to the location and spread of tumors (i.e. stage Tis, Ta and T1-4; Fig. 1), and how to directly against superficial bladder cancer is a key issue that must be resolved in order to improve disease prognosis [28–31].

Determining the appropriate course of treatment depends on the stage of bladder cancer [32, 33]. In the case of non-invasive bladder tumors, the gold standard of primary therapy is transurethral resection of the bladder tumor (TUR-BT), which allows the bladder to retain functionality; however, TUR-BT commonly results in tumor relapse [21]. This has led to the widespread use of Bacillus Calmette–Guérin (BCG) [34] or chemotherapy agents [35], such as Mitomycin C, Adriamycin, Epirubicin or Thiotepa, as adjuvant therapies through intravesical instillation. Chemical agents [36–38], such as Mitomycin C, can restrain the DNA, RNA, and proteins to suppress the proliferation of cancer cells. However, these agents have a number of negative side effects, such as chemical cystitis and other irritating symptoms. Furthermore, long-term chemical cystitis can lead to bladder contraction and a reduction in functionality. Patients suffering from unfavorable differentiation associated with the recurrences of cancer are generally administered intravesical therapy in the form of immune agent BCG [39, 40]. Adverse reactions to these chemical therapies include cystitis (67 %), fever (25 %), haematuria (23 %), and/or increased frequency of urination (71 %) [41]. In some cases, the use of these treatments has been associated with severe toxicity, leading to septicemia, disseminated intravascular coagulation, and multiple organ failure, which can reduce a patient’s desire to be treated [42–44].

Despite the fact that BCG is currently the most commonly used intravesical therapy in superficial bladder cancer treatment, many studies have reported that this agent is only able to delay early recurrence [45]. Furthermore, the widespread use of BCG and chemotherapy causes bladder cancer patients to suffer from high rates of recurrence and rapid disease progression [46, 47]. Thus, compared with other instillation agents, BCG appears somewhat limited in its therapeutic effectiveness [48]. Conversely, nanoparticle technology, which has been used in recent years, has shown great promise in increasing the efficacy of drugs and preventing adverse reactions. The field of medicine stands to benefit significantly from advances in nanotechnology, specifically from improvements in detection imaging and tumor therapy [49]. Nanoparticle technologies were developed to be controlled drug delivery systems with unique targeting for cancer treatment [50]. Many kinds of delivery systems have been developed for bladder cancer therapy using different materials and types of nanoparticles, such as metal/gold, polymeric, liposome and lipid, and protein nanoparticles.

## Application of Nanoparticles in Urinary Bladder Cancer

### Metal/Gold Nanoparticles

Metal nanoparticles are widely used in engineering as well as biomedical sciences [51]. These include magnetic nanoparticles “Fe3O4, Fe-Au alloy” [52, 53] as well as gold [54] and silver [55] nanoparticles, which may be conjugated with antibodies, ligands, or other drugs in order to modify functional groups [56]. Important developments are being made in the field of nanotechnology for applications in magnetic separation, the enrichment of the target analytes, targeted drug delivery, targeted gene delivery, and diagnostic imaging. Important imaging modalities which aid in the visualization of disease states, include MRI, CT, PET, ultrasound, SERS, and optical imaging [57–61].

Because of outstanding biocompatibility, gold was among the first metallic biomaterial to be developed [62]. Furthermore, gold nanoparticles (GNPs) have strong spectral absorption properties when the diameter of the gold particle is smaller than the wavelength of the incoming ray [63]. When nanoparticles absorb energy at a specific wavelength, conduction band electrons from the surface of the particle become polarized and produce instantaneous dipole forces, leading to coherent dipole oscillation in a phenomenon referred to as surface plasma resonance (SPR) [64–66]. Various factors affect the properties of SPR, such as size, shape of the nanoparticles as well as other variables related to chemical structure. The absorption wavelength presents a non-linear red shift associated with the diameter or aspect ratio of the gold nanoparticles.

Laser induced thermotherapy involves the use of a laser to induce heat in tissue, which in-turn leads to coagulative necrosis that destroys tumor cells [67–69]. Plasmonic photothermal therapy (PPTT) applies the optical properties of SPR to assist laser-induced thermotherapy. This technique uses GNPs to enhance the effects of target therapy [69, 70]. Specifically, GNPs absorb specific wavelengths of light suitable for the generation of thermal energy while enhancing spatial selectivity in the application of hyperthermia therapy. One novel adjuvant therapy based on heat effects involves the use of specially designed nanomaterials with high photothermal conversion capability, such as nanospheres, nanoshells, nanorods, nanocages [69]. This treatment provides obvious benefits even after a short treatment time and achieves a hyperthermic state with relatively low laser power, thereby avoiding injury to adjacent healthy tissue.

GNPs provide excellent biocompatibility, modulability, and optical properties [71, 72]. In addition, GNPs are able to modify particular nucleic acids and protein molecules to facilitate the rapid detection of abnormal genes or cancer cells, which makes it possible to diagnose diseases more quickly and easily [73]. The superior photothermal properties of GNPs, compared with other nanoparticles (e.g. core–shell silica nanoparticles, magnetic nanoparticles, cerium oxide “CeO2, TiO2, ZnO”, and quantum dots), has resulted in a gradual shift from these materials to the use of GNPs [69]. GNPs also provide excellent chemical stability and a strong affinity to biomolecules, which facilitates the detection and treatment of cancer. Indeed, the biocompatibility and non-cytotoxic properties of GNPs have the greatest potential for future clinical applications. Thus far, GNPs have been most widely applied in the treatment of breast cancer, oral cavity cancer, lung cancer, cervical and brain cancer [74–76]. Indeed, results from previous studies have revealed that GNPs offer obvious therapeutic benefits to cancer patients. For example, in one study, exposure to a laser was shown to damage cancer cells; however, GNPs require only half the laser power of regular laser treatment [69]. Combining particles with antibodies or proteins that target the overexpressed antigen in the tumor has also been shown to enhance the therapeutic benefits [69].

**Fig. 2:**
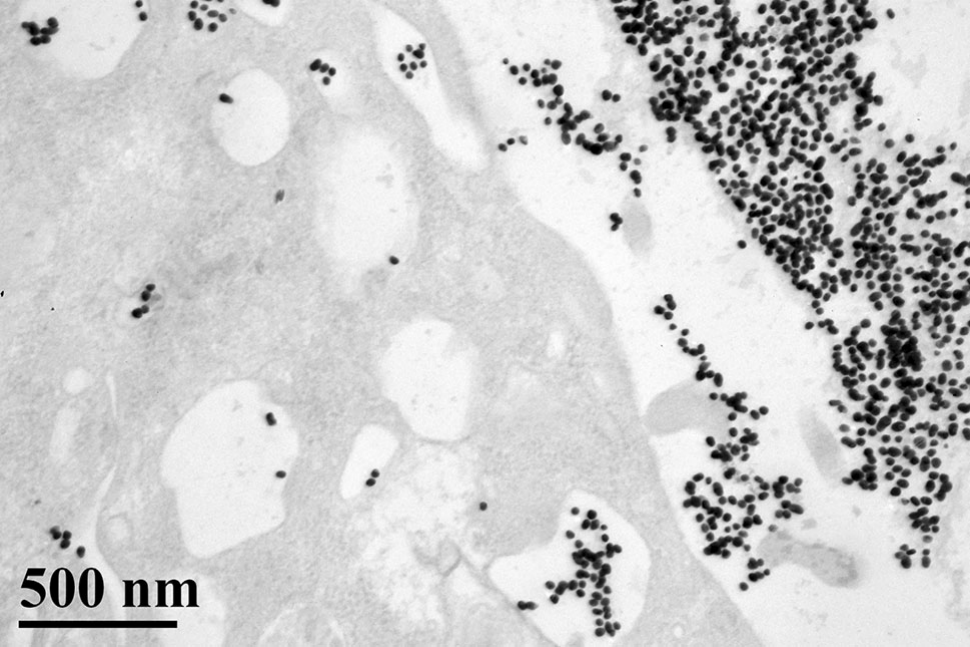
TEM (×60,000) illustrates that the bladder cancer cell is targeted by antibody (anti-EGFR) modified GNPs. Endocytosis is noticed

Previous studies have reported the application of modified gold nanoparticles in bladder cancer. These modified nanoparticles include gum arabic-coated radioactive [77], hyaluronic acid functionalized fluorescent [54], epigallocatechin-3-gallate [75], and antibody-coated silica nanoshells [78, 79]. To destroy tumor cells and preserve normal cells, a targeting system is required. In target therapy, monoclonal antibodies serve as the aiming system of nanoparticles. For different cancer cells, individualized targets are chosen according to the expression of antigens, such as transferring, Her-2, and epidermal growth factor receptor (EGFR) in breast cancer [80–82] and EGFR in oral and epidermal cancers [83, 84]. Globally, bladder cancer is a common cause of carcinomatosis. To target bladder cancer, EGFR, mucin 7, and cytokeratin 20 are commonly used [85–87]. Figure 2, the TEM, shows the bladder cancer cell is targeted by antibody modified GNPs and some endocytosis is found.

### Polymeric Nanoparticles

Polymeric nanoparticles can be made from a wide range of polymers, including natural or synthetic substances composed of macromolecules such as “poly(lactide-coglycolide)”, “poly(lactic acid)”, “poly(ε-caprolactone)”, “chitosan”, and “poly(alkyl cyanoacrylates)” [88–91]. However, many polymeric nanoparticles are toxic to patients; therefore, improving biocompatibility and reducing cytotoxicity of polymeric nanoparticles is imperative for biomedical applications. The composition of polymeric nanoparticles can be varied for the delivery of specific drugs to the surface of specific cells [91]. The first step in using a polymeric carrier is the design a polymeric structure that is biodegradable to ensure that they retain their properties in vivo only for as long as needed. Specifically, biodegradability ensures that polymeric carriers degrade into small molecules that can be metabolized and excreted from the body. Previous studies on the treatment of bladder cancer with drugs formulated by polymeric nanoparticles have shown considerable promise [92, 93]. Compared with other drug delivery systems, polymeric nano-carriers are easier to synthesize, less expensive, and provide superior biocompatibility and biodegradability. They are also non-immunogenic, non-toxic, and water soluble.

### Liposome and Lipid Nanoparticles

Liposomes are artificially-synthesized mono-layer or bi-layer phospholipid vesicles, which have been developed for the transport of molecules, such as drug molecules, nucleotides, protein, and plasmids [94, 95]. Previous studies have indicated that large negatively-charged multilamellar vesicles improve binding affinity and increase the inhibition of four various human bladder tumor cell lines: 253J, J82, T24, and TCCSUP [96, 97].

Oncogene overexpression is one of the major causes of urothelial carcinoma; therefore, the silencing of oncogenes via small interfering RNA (siRNA) coated with liposomes may provide an effective approach to the prevention of bladder cancer [98, 99]. Moreover, the intravesical instillation of liposomes encapsulated with cytotoxic agents has been found to improve the efficacy of intravesical therapies used in the treatment of bladder cancer [100, 101]. Indeed, one highly feasible treatment modality involves intravesical administration of plasmid-containing liposomes, such as IL-2 [102, 103], IL-4 [104], IL-12 [105], interferon-gamma [106], and granulocyte macrophage colony-stimulating factor [107]. Furthermore, in a number of clinical trials, it has been found that intravesical liposomes have similar therapeutic efficacy and can improve the pain score of patients without unanticipated adverse effects [108, 109].

Two lipid nanoparticle systems have previously been applied in cancer therapy: solid lipid nanoparticles (SLNs) and nanostructured lipid carriers (NLCs), both of which are composed of lipids instead of phospholipids [110–112]. SLNs are prepared from a single purified lipid and forma crystalline lattice which allows the incorporation of small molecular drugs. NLCs allow a mixture of lipid types to create a lipid matrix as imperfect as possible. Because of their unique size dependent properties, SLNs are at the forefront of the rapidly developing field of nanotechnology, with numerous potential applications in drug delivery [113]. The ability to incorporate drugs into nanocarriers offers a new vehicle for drug delivery that could be used to improve drug targeting. Indeed, over the past few years, nanostructured lipid carriers have been attracting considerable interest as alternative carriers for anticancer pharmaceuticals. However, many anticancer mixtures are limited with regard to solubility and specificity and are toxic to normal tissue [114]. These mixtures are also associated with poor specificity and steadiness, pharmaceutical resistance, rapid degradation, the need for large-scale output procedures, a fast release of the pharmaceutical from its carrier scheme, the residues of the organic solvents utilized in the output method and the toxicity from the polymer with esteem to the carrier scheme are anticipated to be overcome through use of the nanostructured lipid carrier.

### Protein Nanoparticles

Colloidal drug carrier systems provide selective drug targeting through the use of modified protein nanoparticles, which reduces the effects of drug toxicity [78, 115]. Protein materials used in vivo solves the enzyme-induced degradable problem and provides considerable advantages over colloidal carriers, such as liposomes and cell ghosts. They are composed of biological components capable of delivering a range of molecules, both large and small. Indeed, protein nanoparticles have already been employed as pharmaceutical carriers in a number of cancer therapies [97, 116]. For example, protein nanoparticles can be used in the delivery of protein therapeutics to the lung. They can also be incorporated into biodegradable polymer microspheres/nanospheres to facilitate the controlled release depot or oral delivery. Many researchers are currently focused on the preparation of nanoparticles using proteins such as albumin, gelatin, gliadin, and legumin. In intravesical therapy, commercial paclitaxel contains Cremophor, which can cause micelle formation and interfere with the transportation of paclitaxel across the urothelium. To improve the delivery of paclitaxel in intravesical therapy against bladder cancer, Lu et al. [117] developed a paclitaxel-loaded gelatin protein nanoparticle. Results from that study as well as other research have demonstrated the potential of protein nanoparticles as drug delivery systems for parenteral, peroral, and ocular administration, and may also be a vaccine adjuvant.

## Discussion and Conclusions

The aim of using nanoparticles in cancer treatment is to increase drug specificity and thereby improve treatment outcomes. Indeed, the unique properties of metallic, polymeric, lipid, and protein nanoparticles have been shown to provide considerable benefits in the treatment of superficial urothelial cancer. Intravesical drug delivery is superior to oral therapy in the treatment of bladder cancer, which enables the administration of drugs directly to bladder lesions and reduces the risk of systemic side effects [118]. However, intravesical therapy has limited therapeutic efficacy due to the bladder permeability barrier and periodic bladder discharge. Fortunately, the development of nanoparticles as pharmaceutical carriers has helped to overcome many of these disadvantages. Previous evidence (mostly from animal studies) has supported the application of nanotechnology in intravesical therapy through the retention of drugs in the bladder and the enhancement of drug permeability in bladder cancer [117, 119]. Doubtlessly, nanoparticles will continue to play a dominant role in the coming generations of intravesical therapy against bladder diseases. However, to reduce the risk of distant metastasis, the standard treatment for muscle-invasive bladder cancer remains radical cystectomy, as opposed to regional or systemic drug therapy. As a result, the focus in the application of nanotechnology in bladder cancer treatment remains on non-muscle invasive forms of the disease. Adjuvant therapy in conjunction with nanoparticles lowers the rate of recurrence and reduces the risk of negative side effects associated with traditional intravesical chemotherapy and immune therapy [69].

The excellent photothermal properties of GNPs have led to their application in a variety of treatment techniques which target cancer cells [69, 120, 121]. For example, GNPs can be used to rapidly detect abnormal genes or cancer cells, which improves cancer diagnosis and treatment. In addition, GNPs possess good biocompatibility, good modulability, non-cytotoxicity, and highly specific optical properties. These characteristics suggest that GNPs can benefit a far wider range of clinical applications. Furthermore, differences in electrical charges between GNPs and proteins allow antibody fragments to be conjugated with GNPs [69, 83, 122]. Thus, developing an improved nanoparticle system capable of delivering intact drugs or molecules to the urothelium without severe side effects is a worthy goal for future research.
